# Peer Review of “Machine Learning–Based Hyperglycemia Prediction: Enhancing Risk Assessment in a Cohort of Undiagnosed Individuals”

**DOI:** 10.2196/60389

**Published:** 2024-09-11

**Authors:** Fakhare Alam

**Affiliations:** 1 Oakland University, Rochester, MI, United States

**Keywords:** hyperglycemia, diabetes, machine learning, hypertension, random forest

*This is the peer-review report for “Machine Learning–Based Hyperglycemia Prediction: Enhancing Risk Assessment in a Cohort of Undiagnosed Individuals.”*

## Round 1 Review

### General Comments

This paper [1] introduces a machine learning (ML) methodology for predicting hyperglycemia in one of the cohorts taken from a suburban Nigerian region. The authors present the details of the methodology for participant recruitment and screening, data analysis, and selection of ML models.

### Specific Comments

#### Major Comments

1. The introduction and motivation behind the work are well written. However, there is not enough literature done on the ML aspect of noncommunicable disease prediction; please also cite some of the recent work where ML-based methods are used for noncommunicable disease prediction.
2. Before selecting the features, was there any domain expert consulted? If yes, please provide reasoning on some aspect of feature selection.
3. How were the different ML models selected for the experiment? Please elaborate on some selection criteria such as the combination of tree-based models with other ensemble approaches such as random forest.

#### Minor Comments

1. In Table 2, please reduce the decimal precision up to 2 digits.
2. Figure 1 could be improved with a flow diagram to provide better readability and details of each step.

## Abbreviations

ML: machine learning
